# Opicapone as adjunct to levodopa in treated Parkinson's disease without motor complications: A randomized clinical trial

**DOI:** 10.1111/ene.16420

**Published:** 2025-01-10

**Authors:** Joaquim J. Ferreira, Olivier Rascol, Fabrizio Stocchi, Angelo Antonini, Joana Moreira, Guillermo Castilla‐Fernández, José‐Francisco Rocha, Joerg Holenz, Werner Poewe, the Epsilon Study investigators, Emke Marechal, Bruno Bergmans, Mieke De Weweire, Teodora Manolova‐Mancheva, Aleksandar Bosilkov, Lyubomir Haralanov, Ivan Milanov, Rosen Ikonomov, Krasimir Kirilov, Valcho Naydenov, Alim Izmailov, Latchezar Traykov, Marek Balaz, Ladislav Pazdera, Michal Bajacek, Ondrej Skoda, Luisa Bartlova, Radomir Talab, Jakub Hort, Martin Valis, Edvard Ehler, Olivier Rascol, Sophie Drapier, Luc Defebvre, Stephane Thobois, Giovanni Castelnovo, Lars Toenges, Patricia Krause, Bjorn Falkenburger, Johannes Schwarz, Fabian Klostermann, Alfons Schnitzler, Maria Antonietta Volonte, Alessandro Tessitore, Paolo Barone, Carlo Colosimo, Giovanna Calandra Buonaura, Diego Centonze, Maria Francesca De Pandis, Laura Vacca, Angelo Antonini, Ewa Trzebinska‐Frydrychowska, Joanna Siuda, Jerzy Machowski, Jan Ilkowski, Marcin Nastaj, Monika Rudzinska‐Bar, Katarzyna Kasprzyk‐Galon, Mariusz Grudniak, Joaquim J. Ferreira, Miguel F. Gago, Alexandre Mendes, Svetlana Kostic Dedic, Marina Svetel, Zorica Knezevic, Jasmina Jovic, Pablo Mir Rivera, Gurutz Linazasoro Cristobal, Ernest Balaguer Martinez, Tania Delgado Ballestero, Francesc Valldeoriola Serra, Jerzy Krupinski, Nuria Caballol Pons, Matilde Calopa Garriga, Eduardo Aguera Morales, Jorge Hernandez Vara, Raif Cakmur, Hasmet Hanagasi, Ferda Uslu, Okan Dogu, Bulent Elibol, Camille Carroll, Richard Walker, David Ledingham, Esther Sammler, Victoria Marshall, Ihor Pasiura, Nataliia Buchakchyiska, Liudmyla Dziak, Sergii Moskovko, Yanosh Sanotskyy, Olexandr Kozyolkin, Tatyana Slobodin

**Affiliations:** ^1^ IMM – Instituto de Medicina Molecular João Lobo Antunes, Faculdade de Medicina Universidade Lisboa Lisbon Portugal; ^2^ CNS – Campus Neurológico Torres Vedras Portugal; ^3^ Clinical Investigation Centre CIC1436, Department of Neurosciences and Clinical Pharmacology, Centre of Excellence for Neurodegeneration COEN NeuroToul, and NS‐Park/FCRIN Network University of Toulouse 3, University Hospital of Toulouse, INSERM Toulouse France; ^4^ University San Raffaele Roma and Institute for Research and Medical Care IRCCS San Raffaele Rome Italy; ^5^ Parkinson and Movement Disorders Unit, Centre for Rare Neurological Diseases (ERN‐RND), Department of Neuroscience University of Padua Padua Italy; ^6^ BIAL – Portela & C^a^ S.A Coronado Portugal; ^7^ BIAL R&D Investments Coronado Portugal; ^8^ Department of Neurology Medical University of Innsbruck Innsbruck Austria

**Keywords:** clinical trial, COMT, levodopa, opicapone, Parkinson's disease

## Abstract

**Background:**

Catechol‐O‐methyl transferase (COMT) inhibitors are routinely used to manage motor fluctuations in Parkinson's disease (PD). We assessed the effect of opicapone on motor symptom severity in levodopa‐treated patients without motor complications.

**Methods:**

This was a randomized, double‐blind, 24‐week, placebo‐controlled study of opicapone 50 mg as adjunct to levodopa (NCT04978597). Levodopa‐treated patients without motor complications were randomized to 24 weeks of double‐blind treatment with adjunct opicapone 50 mg or matching placebo. The primary efficacy endpoint was the mean change from baseline to week 24 in Movement Disorder Society‐Unified Parkinson's Disease Rating Scale Part III (MDS‐UPDRS‐III) total score.

**Results:**

A total of 355 patients were randomized (opicapone 50 mg *n* = 177, placebo *n* = 178) and 322 (91%) completed the double‐blind period. The adjusted mean [95% CI] change from baseline to week 24 in MDS‐UPDRS‐III subscore was −6.5 [−7.9, −5.2] in the opicapone group versus −4.3 [−5.7, 3.0] in the placebo group resulting in a significant difference of −2.2 [−3.9, −0.5] favoring opicapone (*p* = 0.010). There was no difference in the incidence of patients who developed motor complications (5.5% with opicapone vs. 9.8% with placebo) and the incidence of adverse events considered related to study medication was similar between groups (opicapone 10.2% vs. placebo 13.5%).

**Conclusions:**

Treatment with once‐daily adjunct opicapone was well tolerated, improved motor severity, and did not induce the development of motor complications. These results support the clinical usefulness of opicapone in the management of PD patients without motor complications.

## INTRODUCTION

Even when levodopa is administered with a dopa decarboxylase inhibitor (DDCI), peripheral levodopa metabolism is shunted to the catechol‐O‐methyl transferase (COMT) pathway, and less than 10% of levodopa crosses the blood–brain barrier [1]. While not currently included in the approved indication, the use of COMT inhibitors in levodopa‐treated patients without motor fluctuations has been repeatedly discussed for a number of reasons [2]. First, to improve levodopa bioavailability and therefore the magnitude of response and second, to reduce fluctuations in synaptic dopamine levels resulting in a more stable motor benefit in the short and medium term, and potentially delaying the emergence of motor complications (dyskinesia and motor fluctuations) in the longer‐term [2].

The clinical utility of blocking both routes of levodopa metabolism to optimize the magnitude of response *before* the emergence of motor complications has been previously explored. In a 6‐month study with tolcapone, early COMT inhibition significantly improved activities of daily living and motor scores, and fewer patients in the tolcapone groups than in the placebo group developed motor fluctuations during the trial [3]. Similarly a 39‐week study with the levodopa/carbidopa/entacapone (LCE) combination showed a significant difference in total Unified Parkinson's Disease Rating Scale (UPDRS) scores in favor of the COMT inhibitor arm versus traditional levodopa/DDCI therapy [4]. In terms of delaying dyskinesia development, the failure of the STRIDE study to show a benefit with early LCE treatment [5] has been taken to suggest that there is no such advantage to early COMT inhibition. However, pharmacokinetic studies found that the dosing interval was inadequate and that the higher levodopa dose equivalents in the LCE group increased the risk of dyskinesia development [5, 6, 7, 8].

Opicapone is a third‐generation, potent, and selective peripheral COMT inhibitor that has proven generally well‐tolerated and efficacious in reducing OFF‐time in patients with PD and end‐of‐dose motor fluctuations [9, 10, 11]. Administration of opicapone as an adjunct to levodopa increases levodopa plasma bioavailability (area under the curve, AUC) and trough levels (*C*_min_) with lesser effect on peak levels (*C*_max_) [12]. In this study, we hypothesized that the addition of once‐daily opicapone 50 mg would enhance the clinical benefit of oral levodopa/DDCI therapy in PD patients without motor complications.

## METHODS

### Study design and participants

We conducted a randomized, double‐blind, placebo‐controlled, 24‐week study to evaluate the effects of add‐on opicapone in early levodopa‐treated PD patients without motor complications. Adult men or women, aged 30–80 years, were eligible if they had a clinical diagnosis of PD [13] within 5 years, a modified Hoehn and Yahr stage [14] of 1–2.5 (on levodopa therapy) and a Movement Disorder Society sponsored revision of the UPDRS (MDS‐UPDRS) [15] Part III (Motor) score ≥20. Participants had to have been treated with levodopa/DDCI for ≥1 year (with stable dosing of 300–500 mg given 3–4 times daily for ≥4 weeks prior to study entry). They had to be naive of COMT inhibitors and free of motor complications but had to be in need of enhanced motor control as judged by patients and investigators. Key exclusion criteria included non‐idiopathic PD, signs of motor complications as defined by an MDS‐UPDRS Part IV score of >0, previous or planned surgery or deep brain stimulation for PD, or any medical condition that might interfere with assessments, including clinically significant cardiovascular disease or psychiatric illness. Treatment with monoamine oxidase inhibitors (except for selegiline, rasagiline, and safinamide) and anti‐emetics with anti‐dopaminergic action (except domperidone) were prohibited during the study (withdrawn ≥1 month before screening). Full inclusion and exclusion criteria are given in Appendix S1.

Institutional review boards at the 74 participating sites across Europe (Appendix S1) approved the protocol and the trial was conducted in accordance with the Declaration of Helsinki and International Conference on Harmonisation Good Clinical Practice Guidelines. All participants provided written informed consent. The study was registered with Clinicaltrals.gov (NCT04978597).

### Procedures

After a screening period of up to 4 weeks, participants were randomized to 24 weeks of double‐blind treatment with opicapone 50 mg or matched placebo using an Interactive Web Response System (Cenduit IRT) in a ratio of 1:1 to the addition of oral opicapone 50 mg or matching placebo. No additional stratification factors were applied. Site staff, sponsor, and participants were all blinded to treatment allocation and will remain blinded until the end of the ongoing open‐label extension period. Study medication was taken in the evening, ≥1 h after the last dose of levodopa/DDCI. Concomitant stable treatment for PD was permitted, except for COMT inhibitors, and levodopa/carbidopa intestinal gel. No changes to the antiparkinsonian regimen were allowed during the double‐blind period unless adjustment was necessary for participant safety. Post‐baseline assessments were performed at 2 and 4 weeks, and then at 4‐week intervals, either by telephone or at clinic visits (Visits 3, 4, 6, and 9).

### Endpoints

The primary efficacy endpoint was the change from baseline to the end of the double‐blind treatment period in MDS‐UPDRS Part III motor scores. Secondary endpoints included the change from baseline to post‐baseline visits in the MDS total (sum of Parts II + III) and subscores (Parts I, II, IV), the Parkinson's Disease Sleep Scale (PDSS) [16], the Non‐Motor Symptoms Scale (NMSS) [17], and the Parkinson's Disease Questionnaire (PDQ‐39) [18]. In addition, the Wearing‐Off Questionnaire (WOQ‐9) [19] was used to screen for end‐of‐dose symptoms, and the proportion of participants with an improvement at the end of the double‐blind period relative to their baseline condition were assessed using the clinician's and patient's Global Impressions of Improvement (CGI‐I and PGI‐I, respectively) [20]. Levodopa equivalent daily doses (LEDD) were calculated at baseline and end of study using updated formulae [21, 22], including two alternative levodopa conversion factors for opicapone (0.33 and 0.5 [22]) and an LED of 100 mg for safinamide [22].

Treatment‐emergent adverse events (TEAEs), vital signs, and safety laboratory tests were assessed throughout the study. In addition, the Columbia Suicide Severity Rating Scale (C‐SSRS) [23] and the Modified Minnesota Impulsive Disorders Interview (mMIDI) [24] were also assessed.

### Statistical analyses

Based on data from subgroups of patients with early motor fluctuations in previous opicapone studies [9, 10], including a treatment effect of 3 MDS‐UPDRS Part III points between treatment groups with a standard deviation of 8.3 points, we estimated that 162 participants per treatment group would provide ≥90% power to confirm a treatment effect versus placebo. Assuming a screening failure rate of 15%, we planned to screen 382 participants. Analyzed populations included a safety set which included all participants who received ≥1 dose of double‐blind study medication and the double‐blind full analysis set (DB‐FAS) which included all randomized participants who took ≥1 dose of study medication and had ≥1 double‐blind post‐baseline assessment.

The primary efficacy variable was the change from baseline to end of double‐blind treatment in MDS‐UPDRS Part III score and was analyzed in the DB‐FAS using a Mixed Model Repeated Measures (MMRM) approach with fixed effects for baseline, region, randomized treatment, visit, randomized treatment by visit interaction, baseline by visit interaction, as well as participant as a random effect. Spatial Power (SP) structure for the variance–covariance matrix was used. This model used the missing‐at‐random (MAR) assumption, whereby the unobserved missing data were assumed to be like those observed. Continuous secondary efficacy endpoints (MDS‐UPDRS subscores, PDSS‐2 total score, NMSS domain and total scores, PDQ‐39 summary index and subdomain scores) were analyzed in a similar way to the primary efficacy endpoint and the proportion of participants with an improvement in CGI‐I and PGI‐I were analyzed using logistic regression, with randomized treatment and geographical region included in the model. Change from double‐blind baseline to end of week 48 in MDS‐UPDRS Part III and Part IV scores were summarized descriptively. In addition, the percentage of participants with worsening of MDS‐UPDRS Part IV items 4.1 (time spent with dyskinesia) and 4.3 (time spent in OFF state) were summarized.

No adjustments were made for multiplicity as all secondary endpoints were considered exploratory. All hypothesis testing was done using two‐sided alternative hypotheses at the 5% level and were performed using SAS® Version 9.4.

## RESULTS

**FIGURE 1:**
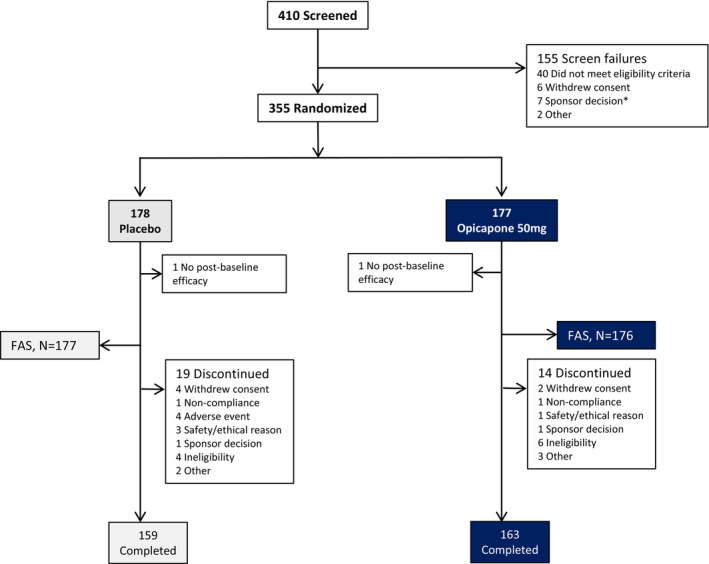
Patient disposition. FAS, full analysis set. *Sponsor decision due to the conflict in Ukraine.

Between May 31, 2021 and July 6, 2022, 410 participants were screened, 355 were enrolled, randomized, and received ≥1 dose of study medication and 322 (90.7%) completed double‐blind treatment (Figure 1). The most common reasons for early discontinuation were protocol deviation and withdrawal of consent. Four participants (2.2%) in the placebo group and none in the opicapone group withdrew during double‐blind treatment due to intolerable adverse events. Baseline characteristics were comparable between groups (Table 1). About 42% of participants in both groups entered the trial on levodopa/DDCI monotherapy. At double‐blind baseline, the mean daily levodopa dose for the DB‐FAS was 386.8 mg in patients randomized to opicapone (LEDD 512.7 mg) and 391.4 mg (LEDD 506.6 mg) in patients randomized to placebo. Adding opicapone to the treatment regimen (calculated at end of study) increased the LEDD in the opicapone arm to 636.0 mg (using a factor of 1.3) and 711.9 mg (using a factor 1.5) versus 510.6 mg in the placebo arm.

**TABLE 1:** Baseline demographics and disease characteristics.

Parameter	Opicapone 50 mg (*N* = 177)	Placebo (*N* = 178)
Age at screening (years), mean (SD)	63.7 (9.5)	64.5 (9.6)
Sex, *n* (%)		
Male	109 (61.6)	121 (68.0)
Female	68 (38.4)	57 (32.0)
Time since PD diagnosis (years), mean (SD)	3.0 (1.2)	2.9 (1.6)
Hoehn and Yahr stage, *n* (%)		
Stage 1	13 (7.3)	8 (4.5)
Stage 1.5	23 (13.0)	17 (9.6)
Stage 2	119 (67.2)	124 (69.7)
Stage 2.5	22 (12.4)	29 (16.3)
MDS‐UPDRS Part III score, mean (SD)	32.7 (10.9)	34.4 (11.7)
Levodopa daily dose (mg), mean (SD)	386.8 (137.2)	391.4 (111.4)
Levodopa equivalent daily dose (mg), mean (SD)^a^	512.7 (191.6)	506.6 (185.8)
Baseline levodopa use, *n* (%)		
Levodopa/DDCI alone	73 (41.2)	75 (42.1)
Levodopa /DDCI and other antiparkinsonian therapy	104 (58.8)	103 (57.9)
Other antiparkinsonian medication, *n* (%)		
Dopamine agonist	75 (42.4)	65 (36.5)
Rasagiline	35 (19.8)	33 (18.5)
Amantadine	16 (9.0)	18 (10.1)

Abbreviations: DDCI, dopa decarboxylase inhibitor; MDS‐UPDRS, Movement Disorder Society‐Unified Parkinson's Disease Rating Scale; PD, Parkinson's disease; SD, standard deviation.

^a^ Levodopa equivalent daily doses were calculated using updated formulae [20, 21].

**FIGURE 2:**
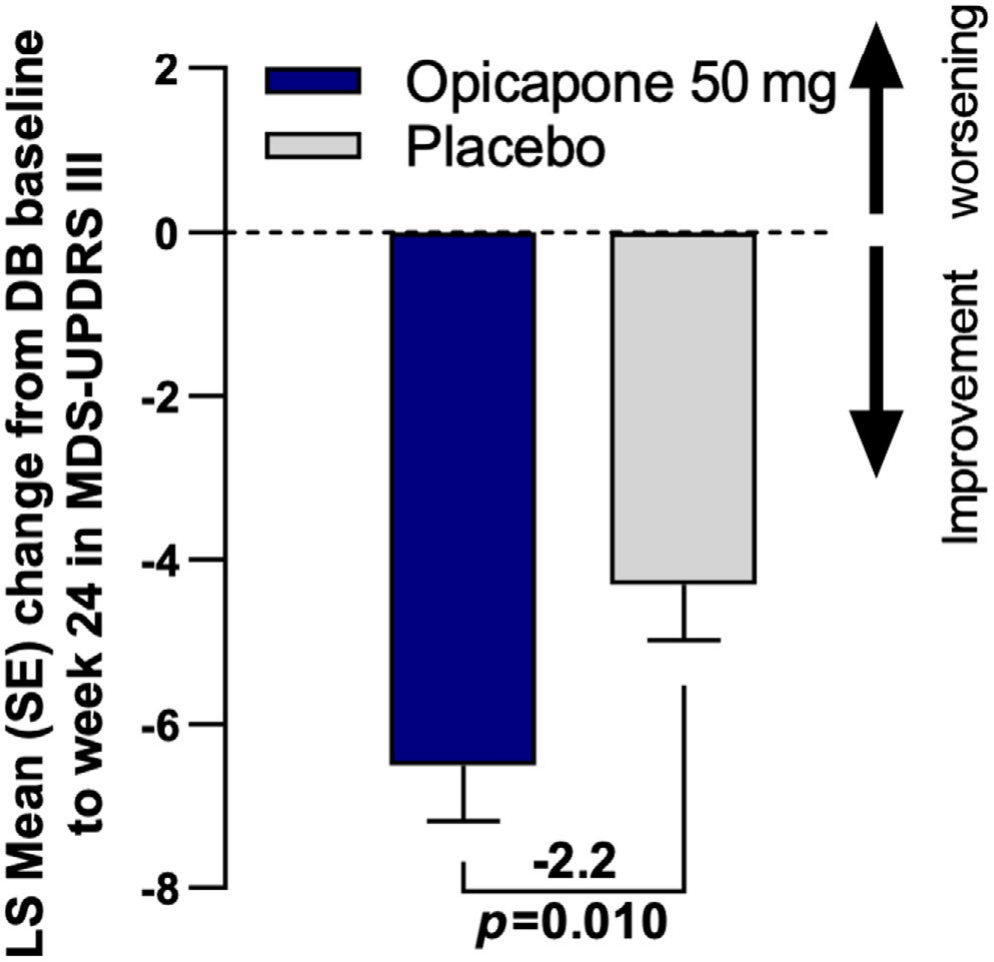
Change from baseline in Movement Disorder Society sponsored revision of the Unified Parkinson's Disease Rating Scale Part III (MDS‐UPDRS‐III) scores at double‐blind (DB) study endpoint. LS, least squares; SE, standard error.

At week 24, the adjusted mean [95% CI] change from baseline in MDS‐UPDRS‐III score in the DB‐FAS opicapone group was −6.5 [−7.9, −5.2] versus −4.3 [−5.7, 3.0] in the placebo group resulting in a significant difference of −2.2 [−3.9, −0.5] favoring opicapone (*p*‐value = 0.010) (Figure 2). Thus, the efficacy hypothesis was met, with opicapone 50 mg showing superiority compared with placebo in improving motor function after 24 weeks of treatment.

Secondary efficacy findings are summarized in Table 2. There were no significant differences between groups in MDS‐UPDRS Parts I and II scores at week 24. Few patients developed motor complications in this study with 5.5% of opicapone‐treated patients and 9.8% of placebo patients shifting to a score >0 on MDS‐UPDRS Part IV; shifts to >0 scores were more common in the placebo group for both item 4.1 (fluctuations) and 4.3 (dyskinesia). The change from baseline to week 24 in MDS‐UPDRS Part IV scores was not significantly different between groups (treatment difference of −0.1 [−0.3, 0.1]; *p* = 0.22) in the DB‐FAS. While the placebo group showed slight worsening of 1.1 (8.3) points on PDSS‐2 total score, participants in the opicapone group were generally maintained at or below baseline levels (small improvement of −0.4 (7.4) points); the difference between groups was significant at week 24 favoring the opicapone 50 mg group (−1.5 [−2.9, −0.1]; *p* = 0.039). There were no significant differences between groups in NMSS total and PDQ‐39 summary index scores. Although there were small shifts in the percentage of participants reporting end‐of‐dose symptoms on the WOQ‐9, the changes were generally small.

**TABLE 2:** Secondary efficacy results.

Parameter	Opicapone 50 mg (*N* = 176)	Placebo (*N* = 177)
MDS‐UPDRS Part I		
Mean (SD) baseline score	6.6 (4.6)	6.8 (5.0)
LS mean [95% CI] change from baseline at week 24	0.4 [−0.2, 1.0]	0.2 [−0.4, 0.7]
LS mean [95% CI] treatment difference		0.2 [−0.5, 0.9]
*P* value (opicapone vs. placebo)		*p* = 0.51
MDS‐UPDRS Part II		
Mean (SD) baseline score	9.1 (5.8)	9.1 (6.1)
LS Mean [95% CI] change from baseline at week 24	−0.4 [−1.1, 0.2]	0.3 [−0.4, 0.9]
LS mean [95% CI] treatment difference		−0.7 [−1.5, 0.2]
*P* value (opicapone vs. placebo)		*p* = 0.12
MDS‐UPDRS Part IV		
Mean (SD) baseline score	0.0 (0.0)	0.0 (0.4)
LS mean [95% CI] change from baseline at week 24	0.3 [0.1, 0.4]	0.4 [0.3, 0.6]
LS mean [95% CI] treatment difference		−0.1 [−0.3, 0.1]
*P* value (opicapone vs. placebo)		*p* = 0.22
*n* (%) patients with worsening of time spent with dyskinesia	2 (1.4%)	5 (3.5%)
*n* (%) patients with worsening of time spent in OFF state	6 (4.1%)	9 (6.3%)
MDS‐UPDRS Part II + III		
Mean (SD) baseline score	41.8 (15.1)	43.5 (16.1)
LS mean [95% CI] change from baseline at week 24	−7.4 [−9.4, −5.4]	−4.6 [−6.6, −2.6]
LS mean [95% CI] treatment difference		−2.8 [−5.4, −0.2]
*P* value (opicapone vs. placebo)		*p* = 0.036
PDSS‐2		
Mean (SD) baseline score	12.4 (8.7)	11.9 (8.6)
LS mean [95% CI] change from baseline at week 24	0.0 [1.2, 1.1]	1.4 [0.3, 2.5]
LS mean [95% CI] treatment difference		1.5 [2.9, 0.1]
*P* value (opicapone vs. placebo)		*p* = 0.039
NMSS total score		
Mean (SD) baseline score	19.9 (17.2)	19.2 (17.3)
LS mean [95% CI] change from baseline at week 24	−1.4 [−3.3, 0.4]	0.5 [−1.4, 2.4]
LS mean [95% CI] treatment difference		−2.0 [−4.4, 0.4]
*P* value (opicapone vs. placebo)		0.102
PDQ‐39 total score		
Mean (SD) baseline score	16.4 (12.9)	15.6 (13.7)
LS mean [95% CI] change from baseline at week 24	0.4 [−0.8, 1.6]	0.7 [−0.5, 1.9]
LS mean [95% CI] treatment difference		0.3 [−1.8, 1.3]
*P* value (opicapone vs. placebo)		*p* = 0.73
PGI‐I		
Participants with improvement, *n* (%)	84 (57.9)	67 (45.9)
Odds ratio for improvement with opicapone vs. placebo		1.7 [1.1, 2.7]
*P* value (opicapone vs. placebo)		*p* = 0.026
CGI‐I		
Participants with improvement	73 (50.3)	67 (46.2)
Odds ratio for improvement with opicapone vs. placebo		1.2 [0.7, 1.9]
*P* value (opicapone vs. placebo)		*p* = 0.49

Abbreviations: CI, confidence interval; CGI‐I, Clinician's Global Impressions of Improvement; LS, least squares; MDS‐UPDRS, Movement Disorder Society‐Unified Parkinson's Disease Rating Scale; NMSS, Non‐Motor Symptoms Scale; PDQ‐39, Parkinson's Disease Questionnaire; PDSS, Parkinson's Disease Sleep Scale; PGI‐I, Patient's Global Impressions of Improvement; SD, standard deviation.

The beneficial effects of opicapone were reflected in the participants’ self‐reported impression of change; at week 24, a significantly higher proportion of opicapone‐treated participants self‐reported an improvement in their clinical condition (58% vs. 46% in placebo, as assessed by PGI‐I). The mean [95% CI] odds ratio for improvement with opicapone versus placebo was 1.70 [1.06, 2.73] (*p* = 0.026). There was no significant difference in the proportion of opicapone‐treated participants rated as improved by their clinician versus placebo (50% vs. 46%, respectively).

Just under half of the participants in each group experienced ≥1 TEAE during the double‐blind period (Table 3), which were usually mild or moderate in intensity. Overall, the incidence of TEAEs considered related to study medication was low and similar between groups (opicapone 10% vs. placebo 14%), and no treatment‐related TEAEs were reported to have a ≥2% difference between groups. Nine participants (5%) in the opicapone group and five participants (3%) in the placebo group experienced serious TEAEs, only one of which was considered to be related to the study treatment (psychotic symptom in the placebo group). Four participants (1%) died due to TEAEs (opicapone *n* = 1, placebo *n* = 3) none of which were considered related to study treatment. Discontinuations due to TEAEs were more frequent for placebo than for the opicapone group (4% vs. 1%), and no TEAE leading to discontinuation was reported for >1 participant in either group.

**TABLE 3:** Summary of treatment‐emergent adverse events.

Parameter	Opicapone 50 mg	Placebo
	*(N* = 177)	*(N* = 178)
	*n* (%)	*n* (%)
TEAE	84 (47.5)	84 (47.2)
Serious TEAE	9 (5.1)	5 (2.8)
Death	1 (0.6)	3 (1.7)
TEAE leading to study discontinuation	2 (1.1)	7 (3.9)
Mild to moderate	83 (46.9)	80 (45.0)
Treatment‐related	18 (10.2)	24 (13.5)

TEAE	Cumulative up to end of DB period in ≥3% of patients	Ongoing at end of DB period	Cumulative up to end of DB period in ≥3% of patients	Ongoing at end of DB period
COVID‐19	15 (8.5)	–	8 (4.5)	1 (0.6)
On and off phenomenon	8 (4.5)	1 (0.6)	5 (2.8)	1 (0.6)
Back pain	8 (4.5)	2 (1.1)	2 (1.1)	–
Tremor	2 (1.1)	2 (1.1)	7 (3.9)	2 (1.1)

Treatment‐related TEAE	Cumulative up to end of DB period in ≥1% of patients	Ongoing at end of DB period	Cumulative up to end of DB period in ≥1% of patients	Ongoing at end of DB period
On and off phenomenon	4 (2.3)	–	3 (1.7)	–
Dyskinesia	2 (1.1)	–	3 (1.7)	2 (1.1)
Nausea	2 (1.1)	–	1 (0.6)	–
Fatigue	2 (1.1)	2 (1.1)	1 (0.6)	–
Tremor	1 (0.6)	1 (0.6)	2 (1.1)	1 (0.6)
Hypotension	1 (0.6)	1 (0.6)	2 (1.1)	–
Orthostatic hypotension	2 (1.1)	2 (1.1)	–	–
Dry mouth	2 (1.1)	1 (0.6)	–	–
Fall	2 (1.1)	–	–	–
Nightmare	–	–	2 (1.1)	–

Abbreviations: DB, double‐blind; TEAE, treatment‐emergent adverse event.

No clinically relevant trends were observed in the opicapone group for clinical laboratory including hematology, serum chemistry, or urinalysis. No trends were observed in other safety assessments, including vital signs, physical and neurological examination, clinical chemistry (including liver enzymes), electrocardiogram (ECG) parameters, C‐SSRS, and mMIDI.

## DISCUSSION

In this randomized, 24‐week, clinical trial, adjunctive treatment with opicapone significantly improved motor impairment without inducing the development of motor complications in levodopa‐treated patients with early PD. The relevance of this motor benefit to patients was highlighted by the significantly greater proportion of patients who self‐reported an improvement in their condition relative to baseline. Additionally, the significant difference in PDSS‐2 total score indicates less nocturnal sleep disturbance with opicapone, likely due to better management of nocturnal bradykinesia and other nocturnal motor symptoms. Other secondary and exploratory measures, including the MDS‐UPDRS Part IV assessment of motor complications, did not find any differences between groups at 24 weeks. Treatment with opicapone was found to be well tolerated, with a safety profile that was similar to placebo and generally more favorable than observed in previous studies in patients with motor fluctuations [25].

The enhanced symptomatic efficacy with opicapone is consistent with the observation that dual inhibition of levodopa peripheral metabolism with a DDCI and a COMT inhibitor increases central bioavailability of levodopa to a greater extent than DDCI alone [1, 2]. The clinical relevance of the effect was shown in the significantly higher proportion of patients who self‐reported improvement with opicapone than with placebo. The discrepancy between the PGI‐I and CGI‐I was also noted in the FIRST‐STEP study of levodopa/carbidopa/entacapone in stably treated patients [4], and may reflect some subjective improvements that the patient appreciates but which are not picked up by standard rating scales nor by the clinician. The change from baseline to end of the double‐blind period in MDS‐UPDRS Part III score of −6.5 points is above both the proposed minimal clinically relevant difference (MCRD) of −3.25 points versus baseline (MDS‐UPDRS) as estimated in clinical practice [26] and also above the MCRD of −5 points versus baseline (UPDRS Version 3) estimated in patients with early PD in clinical trials [27]. Using the current study data, anchored to PGI‐I and CGI, and using two previously described methodologies [28, 29] we estimate the MCRD for MDS‐UPDRS motor scores to range between 4.7 and 6.3 points (depending on the methodology used, see Appendix S1). While the MCRD versus placebo has not yet been evaluated for the MDS‐UPDRS, previous work with UPDRS Version 3 suggested a 2.4‐point change in motor scores could be relevant versus placebo in early PD [29].

In this study, the enhanced bioavailability of levodopa with opicapone did not increase the risk of motor complications and, to the contrary, appeared to be associated with a trend towards reduction of their emergence. Overall, 9.8% of patients in the placebo group and 5.5% in the opicapone group shifted to having a score >0 on the MDS‐UPDRS Part IV, both of which are lower than the rates of wearing‐off (17%) and dyskinesia (6%) development in the FIRST‐STEP study [4]. The lower emergence of motor complications in our study may reflect the shorter study duration, the method of assessment (raters in the FIRST‐STEP study asked specific questions to probe for the emergence of motor complications), and differences in study population. Other studies with separate entacapone have shown that the addition of a COMT inhibitor to the levodopa regimen in subgroups of stably‐treated patients maintained levodopa dose levels over 6 months (whereas patients receiving adjunct placebo had increased levodopa dosing) [30, 31]. The present study design did not allow for any dosing alterations, but since total levodopa dose is considered an important predictor for the development of motor complications [8], such observations may be important in the longer term. The ongoing extension phase, during which PD medication changes are permitted, will provide further opportunity, albeit open‐label, to explore the development of motor complications over 1 year.

The addition of opicapone was well tolerated with a safety profile similar to placebo and generally more favorable than previous studies in patients with motor fluctuations [25]. For example, there were few (<2%) cases of constipation (vs. 5.7% in the pooled pivotal trials [25]) and only four cases (2.3%) of insomnia (vs. 5.1% in the pooled pivotal trials [25]). Rates of discontinuation due to TEAEs were also low and were more frequent with placebo than in the opicapone group (3.9% vs. 1.1%). No new safety signals were reported in this study that could be of clinical concern.

Strengths of this study lie in its randomized, double‐blind evaluation in the early stably‐treated population, which has hitherto been underexplored in PD trials. The 6‐month follow‐up, beyond the minimum duration needed to detect a short‐term symptomatic effect, is another strength of this trial design. The study was specifically designed to evaluate the potential of adding a COMT inhibitor for symptomatic motor benefit and was not adequately powered to detect change in any of the secondary endpoints, all of which were considered exploratory and for which there was no correction for multiplicity. The study was neither designed nor powered to show any effects on reducing or delaying the emergence of motor complications. As demonstrated by the STRIDE study, such a study would necessarily need to be very large and at least 2–4 years’ long [2, 5]. The absence of change in NMSS total score is in line with previous studies and confirms that specific scales are needed are required to track changes in individual non‐motor symptoms [32].

In summary, the addition of adjunct opicapone in levodopa‐treated stable PD patients significantly improved motor impairment without increasing the development of motor complications versus placebo. Taken together with its favorable safety and tolerability profile, these findings position opicapone as an appropriate option for enhancing symptomatic efficacy in early levodopa‐treated patients without motor fluctuations.

## AUTHOR CONTRIBUTIONS

**Angelo Antonini:** Conceptualization; investigation; writing – review and editing. **Fabrizio Stocchi:** Conceptualization; investigation; writing – review and editing. **José‐Francisco Rocha:** Conceptualization; methodology; project administration; writing – original draft; validation. **Guillermo Castilla‐Fernández:** Formal analysis; visualization; validation. **Joaquim J. Ferreira:** Conceptualization; investigation; writing – original draft; validation. **Joana Moreira:** Methodology; project administration; writing – review and editing. **Joerg Holenz:** Methodology; project administration; writing – review and editing. **Olivier Rascol:** Conceptualization; investigation; writing – review and editing. **Werner Poewe:** Conceptualization; investigation; writing – review and editing.

## CONFLICT OF INTEREST STATEMENT

Joaquim J. Ferreira, Olivier Rascol, Fabrizio Stocchi, Angelo Antonini, and Werner Poewe were all investigators in the Epsilon study and report fees for consultancy from BIAL. Joana Moreira, Guillermo Castilla‐Fernández, José‐Francisco Rocha, and Joerg Holenz are employed by BIAL. In addition, Joaquim J. Ferreira has provided consultancy for AbbVie, BIAL, Biogen, Lundbeck, and Sunovion; has received grants from Angelini, Novartis, Medtronic, AbbVie, Zambon, BIAL, Biogen, and Grunenthal; and has received speaker fees for BIAL, Ono, SK Chemical, and Infucure. Olivier Rascol has participated in advisory boards and/or provided consultancy for AbbVie, Adamas, Acorda, Addex, AlzProtect, ApoPharma, AstraZeneca, Axovant, BIAL, Biogen, Britannia, Buckwang, CereSpir, Clevexel, Denali, INC Research, IPMDS, Lundbeck, Lupin, Merck, MundiPharma, NeurATRIS, NeuroDerm, Novartis, ONO Pharma, Osmotica, Parexel, Pfizer, Prexton Therapeutics, Quintiles, Roche, Sanofi, Servier, Sunovion, Theranexus, Takeda, Teva, UCB, Vectura, Watermark Research, XenoPort, XO, and Zambon; and has received grants from Agence Nationale de la Recherche (ANR), CHU de Toulouse, France‐Parkinson, INSERM‐DHOS Recherche Clinique Translationnelle, The Michael J. Fox Foundation, Programme Hospitalier de Recherche Clinique, European Commission (FP7, H2020), and Cure Parkinson's. Fabrizio Stocchi reports honoraria and consulting fees from BIAL, Sunovion, AbbVie, Luosofarmaco, Kjowa, Synagile, Lundbeck, TEVA, UCB, Zambon, Blue Rock, NeuroDerm, Contera, Zambon, Biogen, Ever, and Britannia; speaker fees from BIAL, Sunovion, AbbVie, Luosofarmaco, Kyowa, Synagile, Lundbeck, TEVA, UCB, and Zambon; and travel support from BIAL, Zambon, Synagile, and AbbVie. Angelo Antonini has received compensation for consultancy and speaker‐related activities from UCB, Boehringer Ingelheim, Britannia, AbbVie, Zambon, BIAL, NeuroDerm, Theravance Biopharma, Roche; he receives research support from Chiesi Pharmaceuticals, Lundbeck, Horizon 2020 ‐ Grant 825,785, Horizon2020 Grant 101,016,902, Ministry of Education University and Research (MIUR) Grant ARS01_01081, Cariparo Foundation. He serves as consultant for Boehringer Ingelheim for legal cases on pathological gambling; owns Patent WO2015110261‐A1; and owns shares in PD Neurotechnology Limited. Guillermo Castilla‐Fernández, José‐Francisco Rocha, and Joerg Holenz are employed by BIAL. Werner Poewe has received lecture fees and honoraria for consultancy in relation to clinical drug development programs from AbbVie, AC Immune, Alterity, BIAL, Boehringer, Britannia, Lilly, Eisai, Lundbeck, Roche, Takeda, Britannia, Eisai, Roche, Stada, and Zambon; grant support from The Michael J. Fox Foundation and the EU FP7 & Horizon 2020 programs; and safety monitoring board membership for UCB. He has leadership roles in the Movement Disorder Society, Austrian Society of Neurology, and Austrian PD Society.

## Supporting information


Appendix S1.

## Data Availability

The data that support the findings of this study are available from the corresponding author upon reasonable request.

## Appendix

The Epsilon Study Group: Emke Marechal, Bruno Bergmans, Mieke De Weweire, Teodora Manolova‐Mancheva, Aleksandar Bosilkov, Lyubomir Haralanov, Ivan Milanov, Rosen Ikonomov, Krasimir Kirilov, Valcho Naydenov, Alim Izmailov, Latchezar Traykov, Marek Balaz, Ladislav Pazdera, Michal Bajacek, Ondrej Skoda, Luisa Bartlova, Radomir Talab, Jakub Hort, Martin Valis, Edvard Ehler, Olivier Rascol, Sophie Drapier, Luc Defebvre, Stephane Thobois, Giovanni Castelnovo, Lars Toenges, Patricia Krause, Bjorn Falkenburger, Johannes Schwarz, Fabian Klostermann, Alfons Schnitzler, Maria Antonietta Volonte, Alessandro Tessitore, Paolo Barone, Carlo Colosimo, Giovanna Calandra Buonaura, Diego Centonze, Maria Francesca De Pandis, Laura Vacca, Angelo Antonini, Ewa Trzebinska‐Frydrychowska, Joanna Siuda, Jerzy Machowski, Jan Ilkowski, Marcin Nastaj, Monika Rudzinska‐Bar, Katarzyna Kasprzyk‐Galon, Mariusz Grudniak, Joaquim J. Ferreira, Miguel F. Gago, Alexandre Mendes, Svetlana Kostic Dedic, Marina Svetel, Zorica Knezevic, Jasmina Jovic, Pablo Mir Rivera, Gurutz Linazasoro Cristobal, Ernest Balaguer Martinez, Tania Delgado Ballestero, Francesc Valldeoriola Serra, Jerzy Krupinski, Nuria Caballol Pons, Matilde Calopa Garriga, Eduardo Aguera Morales, Jorge Hernandez Vara, Raif Cakmur, Hasmet Hanagasi, Ferda Uslu, Okan Dogu, Bulent Elibol, Camille Carroll, Richard Walker, David Ledingham, Esther Sammler, Victoria Marshall, Ihor Pasiura, Nataliia Buchakchyiska, Liudmyla Dziak, Sergii Moskovko, Yanosh Sanotskyy, Olexandr Kozyolkin, Tatyana Slobodin.
