# Supplementary material for: Opicapone as adjunct to levodopa in treated Parkinson's disease without motor complications: A randomized clinical trial
Source: Eur J Neurol. 2025 Jan 10;32(1):e16420. doi: 10.1111/ene.16420 (PMC11718218; doi:10.1111/ene.16420)
Supplement: Supplementary file 1 — Appendix S1. [file ENE-32-e16420-s001.pdf]

Supplementary Appendix

Inclusion Criteria
1. Capable of giving signed informed consent
2. 30 to 80 years of age, inclusive, at the time of signing the ICF.
3. Diagnosed with idiopathic PD according to the UK Parkinson's Disease Society Brain Bank Clinical Diagnostic Criteria within the previous 5 years.
4. Disease severity Stages 1 to 2.5 (according to the modified Hoehn & Yahr staging)
5. Signs of treatable motor disability for a minimum of 4 weeks before screening, with minimum threshold with MDS-UPDRS Part III score of ≥20 at both screening and Visit 2, despite stable anti-PD therapy (based on the investigator's judgment).
6. Receiving treatment with levodopa/DDCI (either controlled-release, immediate-release or combined controlled immediate-release) for at least 1 year, and at a stable regimen for at least 4 weeks prior to Visit 2 at a daily dose in the range 300 to 500 mg, 3 to 4 times a day.
7. Naive to COMT inhibitors (including OPC).
8. Male or female.<br>a. A male patient must agree to use contraception and refrain from donating sperm during this period.<br>b. A female patient is eligible to participate if she is not pregnant, not breastfeeding, and at least 1 of the following conditions applies: i) Not a woman of childbearing potential OR agrees to follow the contraceptive guidance
9. Results of the screening laboratory tests are considered clinically acceptable by the Investigator (ie, not clinically relevant for the well-being of the patient or for the purpose of the study).

Exclusion Criteria
1. Non-idiopathic PD (for example, atypical parkinsonism, secondary [acquired or symptomatic] parkinsonism, Parkinson-plus syndrome).
2. Signs of motor complications with a total score of MDS-UPDRS Part IV A+B+C greater than '0' (zero).
3. Treatment with prohibited medication: COMT inhibitors (eg, entacapone, tolcapone), antiemetics with antidopaminergic action (except domperidone) or Duopa™ (carbidopa/levodopa intestinal gel) within the 4 weeks before screening.
4. Concomitant use of monoamine oxidase (MAO-A and MAO-B) inhibitors (e.g. phenelzine, tranylcypromine and moclobemide) other than those for the treatment of PD.
5. Previous or planned (during the entire study duration) deep brain stimulation.
6. Previous stereotactic surgery (eg, pallidotomy, thalamotomy) for PD or with planned stereotactic surgery during the study period.
7. Any investigational medicinal product within the 3 months (or within 5 half-lives, whichever is longer) before screening.
8. Any medical condition that might place the patient at increased risk or interfere with study assessments.
9. Past (within the past year) or present history of suicidal ideation or suicide attempts, as determined by a positive response ('Yes') to either Question 4 or Question 5 on the suicidal ideation portion of the Columbia-Suicide Severity Rating Scale (C-SSRS) (Screening questions).
10. Current or previous (within the past year) diagnosis of psychosis, severe major depression, or other psychiatric disorders that, based on the Investigator's judgment, might place the patient at increased risk or interfere with assessments.
11. A clinically relevant electrocardiogram (ECG) abnormality (relevance should be assessed by a cardiologist if needed).
12. Current evidence of unstable cardiovascular disease, including but not limited to uncontrolled hypertension, myocardial infarction with important systolic or diastolic dysfunction, unstable angina, congestive heart failure (New York Heart Association Class ≥III), and significant cardiac arrhythmia (Mobitz II 2nd or 3rd degree AV block or any other arrhythmia causing hemodynamic repercussions as symptomatic bradycardia or syncope).
13. Prior renal transplant or current renal dialysis.

14. Pheochromocytoma, paraganglioma or other catecholamine secretive neoplasm.
15. Known hypersensitivity to any ingredients of the study treatment.
16. History of neuroleptic malignant syndrome (NMS) or NMS-like syndromes, or non-traumatic rhabdomyolysis.
17. Malignancy within the past 5 years (eg, melanoma, prostate cancer), excluding cutaneous basal or squamous cell cancer resolved by excision.
18. Unstable active narrow-angle or unstable wide-angle glaucoma.
19. History of or current evidence of any relevant disease in the context of this study, ie, with respect to the safety of the patient or related to the study conditions, eg, which may influence the absorption or metabolism (such as a relevant liver disease) of the study treatment.
20. Any abnormality in the liver enzymes (alanine aminotransferase [ALT] and/or aspartate aminotransferase [AST]) >2 times the upper limit of the normal range, in the screening laboratory tests results.
21. Plasma sodium less than 130 mmol/L, white blood cell count less than 3000 cells/mm3, or any other relevant clinical laboratory abnormality that, in the Investigator's opinion, may compromise the patient's safety.

Minimal clinically relevant difference estimations

Method used by Rascol et al [1] (based on Schrag et al [2])

Assumptions of normality of the data were assessed graphically. The mean UPDRS changes from baseline in the different categories of the CGI-I and their 95% confidence intervals were calculated. The MCRD calculated as the mean change of UPDRS Part III score in those patients who were judged to be minimally improved on the CGI-I/PGI-I and results were reviewed in conjunction with the lower limit of the 95% CIs of those judged as unchanged to ensure a plausible MCIC was determined.

<u>Epsilon MCRD based on this analysis:</u>

- Anchored on CGI-I, MCRD of 5.9 vs. baseline.
- Anchored on PGI-I, MCRD of 4.7 vs. baseline.

Method used by Hauser et al [3]

Spearman's correlation coefficients were determined to assess the relationship between the CGI-I/PGI-I and changes in UPDRS Part III scores. Receiver operating characteristic (ROC) curves were developed to calculate cutoff values for UPDRS Part III changes that best distinguished minimal improvement from no change (point on the ROC curve closest to (0,1), calculated as the minimum value of the square root of $(1 – \text{sensitivity})^2 + (1 – \text{specificity})^2$.

<u>Epsilon MCRD based on this analysis:</u>

- Anchored on CGI-I, MCRD of 6.2 vs. baseline.
- Anchored on PGI-I, MCRD of 6.3 vs. baseline.

References

[1] Rascol O (2006) Defining a minimal clinically relevant difference for the unified Parkinson's rating scale: an important but still unmet need. *Mov Disord* **21**, 1059-1061.

[2] Schrag A, Sampaio C, Counsell N, Poewe W (2006) Minimal clinically important change on the unified Parkinson's disease rating scale. *Mov Disord* **21**, 1200-1207.

[3] Hauser RA, Auinger P, Parkinson Study G (2011) Determination of minimal clinically important change in early and advanced Parkinson's disease. *Mov Disord* **26**, 813-818.
